# Difficult Decisions in Minimally Invasive Surgery of the Thymus

**DOI:** 10.3390/cancers13235887

**Published:** 2021-11-23

**Authors:** Ankit Dhamija, Jahnavi Kakuturu, J. W. Awori Hayanga, Alper Toker

**Affiliations:** 1 Department of Cardiothoracic Surgery, Stony Brook University, Stony Brook, NY 11794, USA; ankit.dhamija@stonybrookmedicine.edu; 2 Department of Cardiovascular and Thoracic Surgery, West Virginia University School of Medicine Morgantown, Morgantown, WV 26506, USA; jahnavi.kakuturu@hsc.wvu.edu (J.K.); jeremiah.hayanga@wvumedicine.org (J.W.A.H.)

**Keywords:** thymus, myasthenia gravis, surgery

## Abstract

**Simple Summary:**

The trend for thymoma surgery has been to increase utilization of minimally invasive options for resection; however, the primary objective should be to perform an oncological resection. One must consider the stage of tumor, presence of myasthenia gravis, presence of lymphadenopathy, and size of thymoma prior to deciding optimum surgical strategy.

**Abstract:**

A minimally invasive resection of thymomas has been accepted as standard of care in the last decade for early stage thymomas. This is somewhat controversial in terms of higher-staged thymomas and myasthenia gravis patients due to the prognostic importance of complete resections and the indolent characteristics of the disease process. Despite concerted efforts to standardize minimally invasive approaches, there is still controversy as to the extent of excision, approach of surgery, and the platform utilized. In this article, we aim to provide our surgical perspective of thymic resection and a review of the existing literature.

## 1. Definition of Minimally Invasive Surgery

The thymus has been of interest to surgeons for more than a hundred years. The extent of surgery has ranged from complete resection of thymic, mediastinal and neck fatty tissue, to resection of only tumor itself. The choice of approach to thymic surgery has been subject to constant controversy due to the improvements in visualization and advancement in technology in the last 20 years.

In the early era of surgery, transcervical and transsternal approaches provided satisfactory exposure. Since then, thymic surgery has been described with different incisions, exposures, and equipment. Incisions are named by anatomic location such as transcervical, extended transcervical, right or left thoracic, and subxiphoid. Technology-related approaches using these incisions have included video assisted thoracoscopic surgery (VATS) and robotic options [1]. There are, nevertheless, various combinations of these techniques reported, such as joint VATS and transcervical, joint transcervical and subxiphoid, joint subxiphoid and VATS, and a combination of subxiphoid robotic surgery with bilateral chest incisions. Additionally, the technique of sternal lifting or soft tissue retraction has also been described. To help consolidate the terminology, the International Thymic Malignancy Interest Group (ITMIG) has defined minimally invasive thymectomy as any approach without a sternotomy (including partial sternotomy) or rib-spreading [2].

## 2. Extent of Resection

A simple thymectomy is defined as the complete removal of the thymus, en bloc, without violation of its capsule. When the thymus and thymoma are resected in their entirety, the operation is termed “thymothymectomy”. Where only the thymoma is removed, and thymus gland is left behind, the operation is referred to as a “thymomectomy”. An extended thymectomy includes a thymothymectomy along with resection of mediastinal fatty tissue, pericardial fatty tissue, and bilateral mediastinal pleura en bloc. Moreover, under certain circumstances, a radical or maximal thymectomy may be the procedure of choice. This requires an additional neck incision to access yet more fatty tissue surrounding the carotid artery and trachea. To date, no interinstitutional consensus for surgical management of thymic malignancies exists. In our hands, a complete thymoma surgery refers to the removal of the thymus, thymoma, and fatty mediastinal tissue in its entirety, en bloc [2]. “Shelling out” thymic epithelial tumors (TETs) from the surrounding thymus and fatty tissue is not recommended, as microscopic transcapsular invasion is difficult to detect intraoperatively. Therefore, it is more preferable to obtain a large margin of resection from the perceived extent of the capsule [2]. That being said, there are numerous reports in the field of minimally invasive thymoma surgery claiming successful outcomes of thymoma resection only (thymomectomy). As such, there is ongoing debate regarding this topic [3,4]. 

In early thymomas, Masaoka Stage 1 and 2, the extent of resection should include mediastinal fat and bilateral pleura en bloc [5,6,7]. Thymic surgery is guided by the informal consensus drawn from expert recommendations and guidelines because case-control series and prospective randomized trials are lacking. In 2010, Masaoka himself provided interesting anecdotal reports [8]. By comparing his previous experiences at two different hospitals, he observed that overall survival rates in patients with Stage I and II thymomas undergoing extended thymothymectomy was superior to patients in the thymomectomy series (10-year survival rates of Stage I: 87.1% vs. 66.0%, those of Stage II: 80.6% vs. 60.0%). He attributed survival differences to the surgical oncological techniques used, as he performed different operations at each hospital. 

There are many reasons to perform an extended thymoma resection:

1. Possibility of multicentric thymoma development.
2. Possibility of myasthenia gravis (MG) development in the subsequent years if only a thymectomy is performed. Post-thymectomy MG has been shown to correlate with higher levels of Acetylcholine Receptor antibodies (ARab), which tend to correlate with myasthenic symptoms [9,10]. Regardless of whether ARab levels are elevated or not, our preference is to perform an extended thymothymectomy.
3. To decrease the risk of local recurrences.
4. Control of conditions such as pure red cell aplasia and hypogammaglobulinemia.

## 3. Difficult Decisions and Common Errors

**Figure 1:**
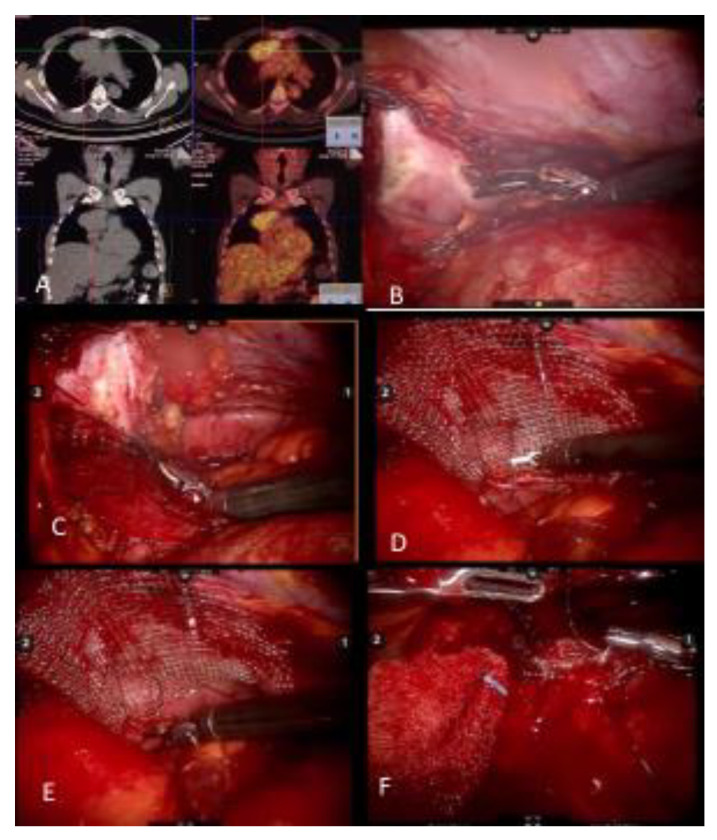
(**A**) Noncontrast CT of a myasthenia gravis patient with a thymoma and PET CT demonstrating FDG uptake. (**B**) Pericardial resection using the robotic bipolar instrument. (**C**) Invasion of pericardium may be deep in the left chest and may require upward traction up the pericardium. It is important to identify and preserve the left phrenic nerve from medial and internal side of the pericardium. (**D**) When reconstructing the pericardium with a graft, the first suture placed allows upward traction of the hilar pericardium. (**E**) A continuous suture technique with barbed sutures is preferred when reconstructing the pericardium. (**F**) As the left hilar pericardium is pulled up, attention should be given to not place the graft too tight as to constrict the heart.

Prior reports claim that extensive pleural adhesions, pericardial adhesions, great vessel involvement, and pericardial involvement are contraindications to minimally invasive thymoma resections [2]. In our experience, however, it is rare for thymic masses to involve the superior vena cava or right innominate vein. On the contrary, left innominate vein involvement is more common. We have also found that complete resection of the left innominate vein is feasible without reconstruction if the patient does not have a history of a radical neck dissection for a neck tumor [11]. Pericardial resection has been previously considered a contraindication to minimally invasive surgery for thymoma, but we have demonstrated technical feasibility despite pericardial involvement, using both the robotic console and VATS approach (Figure 1).

Not only is it important to keep the specimen en bloc upon resection, but for the same reasons it is important to extract the tumor from the patient without perforation or spillage. Therefore, the size of incision necessary to extract the tumor may determine the feasibility of a minimally invasive or open approach. In addition to size, the extent of invasiveness is a factor to consider while deciding surgical approach. Some tumors may be small but are still considered invasive. We have demonstrated that Masaoka Stage I and II thymomas are ideal candidates for minimally invasive thymoma resection and that Masaoka Stage rather than the thymoma size is a more important prognostic factor [12].

## 4. Myasthenia Gravis

Patients with MG are considered separate entities when discussing the nuances of surgical technique. First, the duration of surgery is important for patients with MG whereas patients without MG do not have the same time limitations. Longer operative times potentially increase MG-related postoperative complications such as myasthenic crisis, atelectasis, and pneumonia. Surgeon expertise is one of the most important factors that influences duration of surgery. This becomes increasingly relevant when resection and reconstruction of vital structures may prolong surgical time. Secondly, in patients with Masaoka Stage III, and in some patients with Masaoka Stage II disease, phrenic nerve, lung, pericardial, or pleural resections may be needed. Even when there is no invasion into these structures in Masaoka Stage II, greater experience in minimally invasive techniques may be required to prevent unnecessary collateral damage [13].

The oncological and neurological outcomes of MG patients and thymoma have been studied after robotic surgery [14]. In a series by Romano et al., patients who had their thymus removed showed a complete stable remission (CSR) rate of 14.7% at three years and a clinical improvement in 77% of patients with MG. Conversely, 23.6% experienced either no substantial change or worsened symptoms. The average operation duration was 126 min, and the average hospital stay was 5.1 days [14]. This length of stay (LOS) with robotic surgery is a clear example of the outcome of prolonged surgical time. 

In a prior report, we have demonstrated that the duration of surgery is the single, most important factor for determining next morning discharge in patients with MG who underwent thymectomy [15]. Of the patients discharged the following morning, the mean operative time was 42 min, and the patients with a longer LOS had a mean operative time of 55 min [15]. A recent study published a median LOS of 3 days for minimally invasive thymic surgery and four days for open surgery. When performing thymoma resection in MG patients with a robotic, minimally invasive approach, the LOS has been shown to be even shorter [16]. If enhanced recovery after surgery (ERAS) protocols are applied, open surgery patients have been shown to be discharged within three days. Kumar et al., analyzed outcomes after robotic thymoma resections and found that resection of surrounding structures, conversion to open surgery and postoperative complications were significantly higher in MG patients [17]. In patients with MG and thymoma, the aim should be to decrease the operative time and achieve the highest oncologic quality. The operative approach is left to the discretion of the surgeon and is usually determined by level of expertise and severity of disease. 

## 5. Lymph Node Dissection

Thymoma has been reported as one of the most common anterior mediastinal masses with a present, but very low rate of mediastinal lymph node metastasis. Approximately 90% of the involved nodes are located in the anterior mediastinum and there has been a trend towards removing the anterior mediastinal nodes routinely, as recommended by ITMIG [18]. A more extensive nodal assessment may be indicated in higher stage thymomas [18]. A recently published study discussed the importance of a routine mediastinal and cervical lymph node dissection in particular tumors, such as thymic carcinoids and thymic carcinoma [19].

Kondo and Monden were the first to report the incidence of lymph node positivity and its effect on prognosis in patients with thymoma, thymic carcinoma, and thymic carcinoid [20,21]. The rate of lymphogenous metastasis in thymoma, thymic carcinoma, and thymic carcinoid was found to be 1.8%, 27%, and 28%, respectively [21]. A large database of patients from the United States demonstrated the incidence and prognostic significance of nodal metastases in patients specifically with thymoma as high as 13.3% [22]. In 2013, Park recommended routine resection of all mediastinal lymph nodes in patients undergoing thymic resection, and ITMIG along with the International Association for the Study of Lung Cancer, have proposed a new TNM staging classification emphasizing the importance of lymph node status [19,23]. However, the debate remains regarding benefit of lymphadenectomy [24]. Of note, the mediastinum is a complex part of the thorax. Many histologically different neoplasms may arise from the multiple anatomic structures, and lymph nodes may harbor the metastases secondary to lesions in other parts of the body.

Today, lymph node dissection during minimally invasive surgery may seem unsatisfactory in comparison to open techniques. With the minimally invasive techniques of robotic surgery or VATS, accessible lymph nodes may be limited to the local anterior mediastinum. Therefore, the approach may be guided by the lymph nodes that need to be assessed. A left sided approach will allow access to lymph nodes at the aortopulmonary window and paraaortic region. Similarly, a right-sided approach, will allow access to the right paratracheal region. When performing the subxiphoid approach, both lymph node basins may be accessed. By adding a cervical incision to the subxiphoid approach, a complete lymph node dissection may be possible.

**Figure 2:**
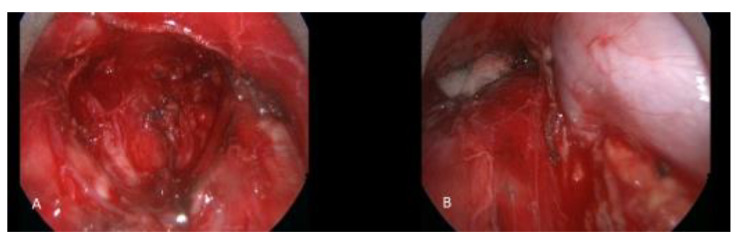
A patient with thymic carcinoma with suspicious mediastinal lymph node metastasis underwent cervical video mediastinoscopy and excision of the mediastinal lymph nodes. (**A**) Subcarinal region after complete removal of lymph node packet. (**B**) Right paratracheal region. The azygos vein and superior vena cava are seen skeletonized after lymphadenectomy.

In our experience, if the final pathologic diagnosis of a thymic tumor performed in a minimally invasive fashion is thymic carcinoma, we subsequently perform a cervical video-assisted mediastinal lymphadenectomy to stage and evaluate for micro-metastatic disease after performing a PET-CT (Figure 2).

## 6. Learning Curve

There is a substantial learning curve in minimally invasive thymoma resections, for both the surgeon and operative team [25,26]. There has been a growing number of thymic resections performed on the robotic platform. The most recent outcome analyses published, are by surgeons who perform primarily robotic resections. Operative times and blood loss were shown to decrease with increasing experience. Additionally, the learning curve for performing a robotic thymectomy is described as 15 to 20 cases [27]. The number of cases needed to comfortably operate in technically demanding situations, such as Masaoka Stage III and larger thymomas, is considerably higher. One commonly discussed parameter of the learning curve is conversion to open surgery. In published series, the conversion rate of robotic surgery ranges from 2% to 15% [28,29,30,31]. These conversions may be avoided if the decision to open is done prior to a major vascular injury or in order to prevent an oncologic safety concern. With experience, surgeons are able to discern the operative approach preoperatively based on radiologic findings.

## 7. Long Term Oncological Outcomes of Minimal Invasive Thymoma Resections

There are several series comparing open and minimally invasive thymoma resections with respect to short term outcomes. Unfortunately, there is yet to be a series that has a long enough follow-up to compare the long term outcomes. A median follow-up of 36–40 months is not considered a long enough period to compare long term survival. According to our experience a minimum follow-up of 60 to 120 months would be appropriate for thymic epithelial tumors. The expected outcomes when using a minimally invasive platform should be comparable to the open approaches. The general expected survival is high, and recurrence is low, as seen by a series in 2003 with 70 patients with Stage I and II thymomas. Of the 70 resected thymomas performed through a multitude of approaches, only two patients recurred (2.8%) and the five-year survival was 91%. All patients who died during the follow-up period were tumor free [32].

Additionally, the long term outcome analysis for thymoma resections, done primarily on the robotic platform, have a limited duration of follow-up. In a series of 23 patients with pathological Masaoka Stage I (21 cases) and II (two cases), no patients showed recurrence at a mean follow-up of 24.8 months [33]. In a propensity score-matched analysis comparing minimally invasive thymoma resection and open surgery, there was no significant difference in margin positivity and five-year survival (89.4% vs. 81.6%) [34]. A systematic review and meta-analysis recently demonstrated that there is no significant difference in the R0 resection rate and locoregional recurrence in patients with Masaoka Stages I and II tumors when comparing minimally invasive thymoma resections versus open thymoma resections [35]. As to prevent recurrence, surgeons should avoid rupture of the capsule and implantation of the tumor to the mediastinum and pleura. Longer follow-up is needed to definitively determine outcomes with open surgery versus minimally invasive surgery.

In a large multi-institutional series with 131 patients, 97.8% were reported alive with a median follow-up of 42 months, ranging from 5 months to 159 months. A pleural recurrence was found in one (0.7%) patient with an original Masaoka Stage IVa tumor. The five-year overall survival rates were 97%, and the five-year thymoma-related survival rates were 100% [36].

Minimally invasive thymoma resections have been reported to decrease early complications and improve the recovery period [36,37]. Despite these findings, minimally invasive thymoma resections have been questioned for long term survival and oncologic completeness. There is a paucity of literature discussing this important question. In an article recently published, the outcomes of robotic thymoma resection were reported to be favorable. The five-year disease specific survival of the patients with thymoma and thymic carcinoma was found to be 100% and 95.2%, respectively [38]. In this series, of the 158 patients who underwent robotic thymic epithelial tumor resections, two patients died of non-thymoma related causes and one patient with thymic carcinoma died due to progression of the disease. Recurrence occurred in nine patients (three liver metastasis, one lymph nodes metastasis, and one bone metastasis). Median follow-up time was 43 months [38]. Minimally invasive surgery for thymoma is expected to have a higher potential risk for pleural dissemination which is attributed to violation of the capsule during the surgical procedure. Additionally, larger and cystic thymomas have higher risk for pleural dissemination [39]. Recurrence rates of thymoma after minimally invasive resection ranges from 0% to 6.7%, and the five-year disease-free survival ranges from 83.3% to 96% [40,41,42,43,44] (Table 1).

A systematic review showed that surgical treatment for Masaoka Stage I and II thymoma has an overall five-year survival rate of 89% to 100% and 71% to 95%, respectively, regardless of the approach [45]. These results were comparable to minimally invasive thymoma resections [42]. In a more recent study, patients with Masaoka Stage I-III demonstrated a 5-year survival of 86.9% for the open group and 90.7% for the minimally invasive group [34].

In all these series, we can see that follow up times are limited for this relatively indolent tumor. Longer follow up is required to make a more definitive suggestion with regards to minimally invasive approaches.

More recently, a South Korean study described long-term outcomes on robotically performed thymoma resections. This study evaluated a broad array of thymic epithelial tumors in multiple stages. The median follow-up was 43 months, which is similar to previously mentioned studies. Thymoma was the most common pathology (*n* = 132), followed by thymic carcinoma (*n* = 24), and neuroendocrine tumors (*n* = 2). Advanced stages were present in 15 patients and were categorized as Masaoka Stage IIIA (*n* = 7), Stage IVA (*n* = 5), and Stage IVB (*n* = 3). Five-year disease specific survival was 100% in thymoma patients and 95% in thymic carcinoma patients. Five-year recurrence free survival was 94% in thymoma resections and 79% in thymic carcinoma resections. Of the 158 total patients, there were nine recurrences seen. Of the 132 thymoma patients, there were only two patients with recurrences, of which one was a Masaoka Stage I and the other Stage IVA. Of the 26 thymic carcinoma patients, there were seven recurrences, and one oftwo2 neuroendocrine tumor resections recurred.

Thymic epithelial masses, especially thymic carcinoma and neuroendocrine tumors, require adjuvant treatment and close follow up from both the surgical and oncology team. In both histopathologic types, lymph node dissection is essential and should be a routine part of the operation. Unfortunately, often the diagnosis of thymic carcinoma or neuroendocrine tumor is not known pre-operatively and the dilemma of having not performed a thorough lymphadenectomy arises. To prevent this dilemma, a high pre-operative SUV uptake on the primary mass or suspicious lymph node activity, should trigger the need for an aggressive lymphadenectomy. In our practice, if dealt with a clinical scenario when the aggressiveness of the tumor is discovered post-operatively, endobronchial ultrasound guided sampling of the mediastinal lymph nodes can be utilized or one can return to the operating room to perform a video assisted mediastinal lymph node dissection. This approach has not been discussed in the literature to our knowledge, and requires further analysis.

## 8. Does Size Matter?

As previously mentioned in the text, the limiting factor to minimally invasive thymoma resection is the invasiveness of the tumor rather than the size. However, as we have stressed above, if required, a large thoracotomy should be performed to remove the thymoma without rupture and spillage. As reported in 2013, the pleural recurrence rate was found to be higher in patients with a thymoma larger than 5 cm after a VATS thymectomy [39]. In this paper, violation of the tumor capsule was the theory behind the higher recurrence rate. Additionally, the Japanese Association for Research of the Thymus (JART) demonstrated that the 10-year recurrence-free survival rate was lower in patients with thymomas larger than 5 cm [46]. Other authors from North America showed there was no correlation between size and completeness of the resection [47]. Complete resection rather than the tumor size has been claimed by others to determine the prognosis in thymic epithelial tumors [48]. Rather than performing a large thoracotomy for safe extraction of the resected thymoma, a subxiphoid approach has been considered. A robotic, subxiphoid approach for thymectomy has shown promise for the treatment of larger thymomas.

In our experience, we have performed robotic thymoma resections via the right, left and subxiphoid approaches. When performing robotic thymectomy through the left chest, surgeons should be cognizant of avoiding injury to the heart upon placement of the trocars. Additionally, the right sided mediastinal fat and right phrenic nerve may be difficult to evaluate via a left thoracic approach, especially in an obese patient. On the other hand, dissecting the left thoracic inlet over the left innominate artery is easier and may reduce incidental injury to the left innominate vein. A right sided approach has been our standard technique, reasons being that the right hemithoracic cavity is larger, it is easier to dock and explore the chest from the right side, and resection of the contralateral pericardiophrenic fatty issue is easier. On the contrary, the distal end of the left innominate vein is more difficult to identify, and it is also here that the left phrenic nerve converges to the midline. Overall, if the tumor is in the right chest cavity or just left to the midline, our preferred approach is through the right thoracic cavity. If it close to the left phrenic nerve, we prefer a bilateral approach, where we begin with the left sided dissection and complete it on the right side. Lastly, if the mass is larger than 5 cm, we prefer a subxiphoid approach.

## 9. Final Comments

It is important to understand that the utilization of the minimally invasive platform for thymoma resection is not a competition for resecting the largest thymoma. Though the approaches are similar, minimally invasive surgery for lung cancer is different than minimally invasive thymoma resection. With a thoracotomy in lung cancer resection, one cannot only address the restrictions due to the thoracotomy itself, but also the loss of lung tissue. In thymoma surgery, there is no loss in lung tissue and a partial sternotomy is not as detrimental as a thoracotomy. Therefore, it is not easy to extrapolate conclusions on thymectomy removal based on data describing and comparing minimally invasive platform being utilized for lung resection.

Below we aim to summarize the risks and benefits of using the minimally invasive platform in thymoma surgery.

### 9.1. Risks

1. Capsule rupture and microscopic implantation of the tumor.
2. Longer duration of operation in patients with Myasthenia Gravis if the surgeon is not experienced in minimally invasive techniques.
3. Inadequate mediastinal lymph node dissection.
4. Masaoka Stage II patients may undergo unnecessary resection of additional organ when performed by inexperienced surgeons.
5. Possible catastrophes such as major vessel injury.
6. May still require a large chest-wall incision to remove the mass.

### 9.2. Benefits

1. Patient threshold to accept minimally invasive surgery as an option is higher.
2. Higher visual and maneuverability quality in experienced hands, especially in the morbidly obese.
3. Aesthetic incisions.
4. Shorter length of stay.

We believe that a surgeon and referring doctor should be aware of the importance of the quality of the surgery. One must consider the balance between risks and benefits described above when considering their approach.

## 10. Conclusions

Surgical approach in thymic surgery remains a contentious issue. There are multiple factors that should be considered, including surgeon experience, presence of MG, Masaoka Stage of tumor, and extent of resection required. Once performed, patients undergoing thymoma resection require long-term follow-up. Regardless of the approach, our recommendation is to perform an extended thymectomy when removing a thymoma. More analyses regarding surgical techniques, minimally invasive approaches, and lymph node dissection for thymoma are necessary to bring us closer to formal consensus.

One important issue is tissue diagnosis. Patients with thymic carcinoma and thymic neuroendocrine tumors do worse than the thymomas. There is a need to differentiate these tumors prior to minimally invasive surgery in order to perform an adequate mediastinal lymph node dissection intraoperatively. Concerning features should be identified with CT, PET/CT or MRI preoperatively. The importance of adjuvant treatment in these patients should not be underestimated.

## Figures and Tables

**Table 1:** Stage I and II Thymoma Resection Survival and Recurrence.

Reference	Number of Patients	Tumor Size (mm)	Follow-Up (Months)	5-Year Survival (%)	Recurrence (*n*)
Liu et al., (2014) [41]	Stage I: 57 Stage II: 19	46 *	44	96.9 (DFS)	2
Ye et al., (2014) [42]	Stage I: 80 Stage II: 45	32.3 ^†^	41	NA	1
Sakamaki et al., (2014) [43]	Stage I: 40 Stage II: 31	35 ^†^	48	92.4 (RFS)	2
Odaka et al., (2015) [44]	Stage I: 33 Stage II: 34	40 ^†^	55	95.8 (DFS)	2
Kang et al., (2021) [38]	Stage I: 126 Stage II: 19	4.6 cm	43 months	93.9 (RFS)	1

*: mean value; ^†^: median values; DFS:5-year disease free survival; RFS: recurrence-free survival; NA: not applicable. Series with median follow up longer than 40 months included.

## Data Availability

No new data were created or analyzed in this study. Data sharing is not applicable to this article.
